# Simple neck pain questions used in surveys, evaluated in relation to health outcomes: a cohort study

**DOI:** 10.1186/1756-0500-5-587

**Published:** 2012-10-26

**Authors:** Anna Grimby-Ekman, Mats Hagberg

**Affiliations:** 1 Department of Public Health and Community Medicine, Sahlgrenska Academy and University Hospital, Box 414, Göteborg, SE-405 30, Sweden

**Keywords:** Musculoskeletal, Neck pain, Validity, Health, Performance, Epidemiological study

## Abstract

**Background:**

The high prevalence of pain reported in many epidemiological studies, and the degree to which this prevalence reflects severe pain is under discussion in the literature. The aim of the present study was to evaluate use of the simple neck pain questions commonly included in large epidemiological survey studies with respect to aspects of health. We investigated if and how an increase in number of days with pain is associated with reduction in health outcomes.

**Methods:**

A cohort of university students (baseline age 19–25 years) were recruited in 2002 and followed annually for 4 years. The baseline response rate was 69% which resulted in 1200 respondents (627 women, 573 men). Participants were asked about present and past pain and perceptions of their general health, sleep disturbance, stress and energy levels, and general performance. The data were analyzed using a mixed model for repeated measurements and a random intercept logistic model.

**Results:**

When reporting present pain, participants also reported lower prevalence of very good health, higher stress and sleep disturbance scores and lower energy score. Among those with current neck pain, additional questions characterizing the pain such as duration (categorized), additional pain sites and decreased general performance were associated with lower probability of very good health and higher amounts of sleep disturbance. Knowing about the presence or not of pain explains more of the variation in health between individuals, than within individuals.

**Conclusion:**

This study of young university students has demonstrated that simple neck pain survey questions capture features of pain that affect aspects of health such as perceived general health, sleep disturbance, mood in terms of stress and energy. Simple pain questions are more useful for group descriptions than for describing or following pain in an individual.

## Background

In epidemiological cohort or surveillance studies, where musculoskeletal pain is only one health aspect among many others investigated, the multidimensional aspects of pain have to be captured in only a few variables. Therefore, multi-item instruments for pain assessment are not suitable for the epidemiological survey setting. In the present paper, we term such assessments ‘simple’, as they only capture simple characteristics of pain, such as its presence or duration [1-8], and are usually dichotomous. They do not address deeper qualities, such as intensity, character, or impact on life.

Assessment of pain is difficult as pain is subjective, multidimensional, and variable in its manifestation and varies over time [9,10]. Chronic musculoskeletal pain has large impact on many aspects of daily life. Several questionnaires have been developed to assess these different dimensions and characteristics of pain (e.g., the pain scales developed by Von Korff et al., the Pain Disability Index (PDI), and instruments of kinesiophobia and fear of pain) [11-15]. The visual analogue scale (VAS), verbal descriptor scales (VDSs), the McGill Pain Questionnaire (MPQ), and similar scales and questionnaires have been developed for assessment of perceived pain intensity, and quality and activity limitations.

Discussions concerning the high prevalence of pain revealed in many epidemiological studies [16-18] led us to evaluate simple pain questions. In intervention studies, or when evaluating treatments in clinical settings, more detailed and complex pain assessments are necessary [19], but these are beyond the scope of the present study.

One important property when evaluating questionnaire-based instruments is its validity, i.e., that it measures what it intended to measure or assess. When evaluating the validity of simple pain questions the focus is here not to question if they assess pain or not, but if the questions assess pain that is of enough severity to be of public health interest. Pain per se is a warning signal we need, but when pain becomes a dysfunctional symptom (e.g. chronic pain, central sensitization, widespread pain…) it is something we want to prevent and cure. In epidemiological studies it may therefore be argued that it is most important to identify pain that affects the life of the individual [9]. However, from the perspectives of work and society, it can be argued that it is most important to identify pain that leads to sick leave, decreased general performance or lower productivity.

Evaluation studies of the Nordic Questionnaire (NQ) are of interest here as they include simple pain questions similar to those investigated in the present paper. Results from studies based on the NQ mostly concern validity with respect to diagnosis, showing mostly that the NQ has high sensitivity and low specificity [20-22], although one study showed that the NQ had high specificity [23]. It is noteworthy to mention that the NQ was never intended to measure diagnosis. Sensitivity should be high, but since severe pain can stem from many causes, other than the specific diagnoses investigated in those studies, low specificity is neither surprising nor a useful measure of quality. However, in one study, good predictive validity was found regarding number of pain sites and association with disability pensioning [24].

The aim of the present study was to evaluate simple pain assessments in relation to the impact on aspects of health (perceived general health, sleep disturbance and mood) and decreased general performance; and whether an increased number of days with pain or additional pain sites are associated with reduced health and decreased general performance.

## Methods

### Data

**Figure 1:**
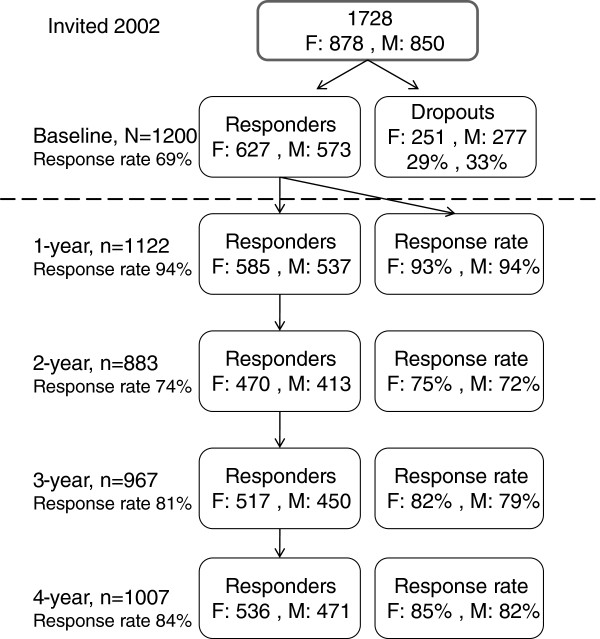
**Participant flowchart showing the time points of data collection.** The response rates are in relation to baseline.

A cohort of university students (baseline age 19–25 years), enrolled in medical and information technology (IT)-related studies, were recruited in 2002 to respond to Internet-based questionnaires providing baseline and further data from four annual follow-ups Figure 1. The baseline response rate was 69% which represented 1200 respondents, 627 women and 573 men. The age of respondents over the 5-year study ranged from 19 to 29 years.

All subjects received written information concerning the study and their right to refuse to participate. The project was approved by the Regional Ethical Review Board at the University of Gothenburg, Gothenburg, Sweden. A more extensive description of the study is presented elsewhere [25].

### Neck pain variables

**Figure 2:**
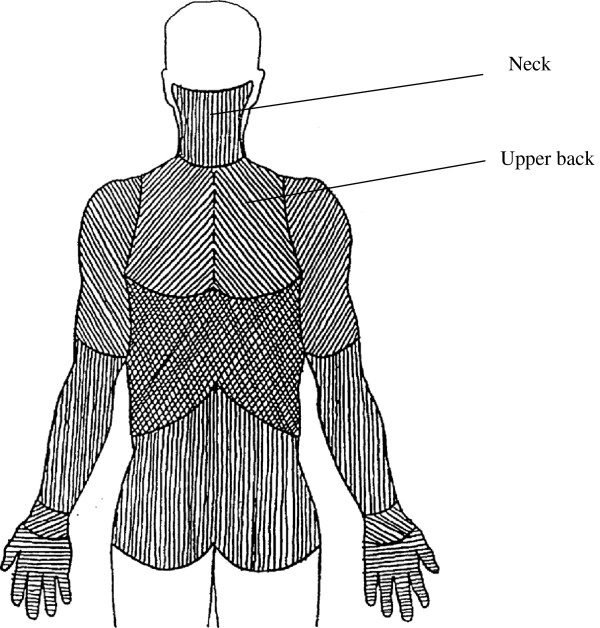
The marked areas ‘neck’ and ‘upper back’ define the variable ‘neck pain’ used in the present paper.

In the present paper neck pain is defined as the two areas ‘neck’ and ‘upper back’ in Figure 2. This is in good agreement with the recommended definition of neck pain according to the work of the Task Force on Neck Pain and Its Associated Disorders [10].

In the present paper, questions were asked about the presence and duration of neck pain and whether the pain decreased the individual’s general performance. The phrasing of the questions, and their possible response values were:

1) *Do you at present have aches*/*pain in the upper part of your back*/*neck*? (0,1)

2) *Number of days with current aches*/*pain* (1,2,3,4, …)

3) *Have you had decreased general performance due to ache*/*pain in muscles or joints over the past 30 days*? (0,1)

Note that the question concerning decreased general performance was not phrased with reference to neck pain, but only to pain in muscles and joints in general. Question a) and b) were also asked for other pain sites (lower back or arms/hands).

### Health outcomes

Selected outcome variables were *perceived general health*[26,27], *sleep disturbance* and *mood* (stress and energy). The variable *general health* was dichotomized according to cutoff points indicated in the results of logistic regressions for ordinal outcomes (cumulative logit). The definition of the dichotomized variable *very good health* is presented in Table 1.

**Table 1:** **Outcome variables**, **questions**, **and responses**

	**VARIABLE**	**QUESTIONS**	**RESPONSE ALTERNATIVES**	**NOTES**
Aspects of health	**Perceived stress**	How do you rate your general state of health?	Very good, pretty good, neither good nor poor, pretty poor, poor	**Very good health**: 1 = Very good health
				0 = Pretty good health or less
	**Sleep disturbance**	During the past 6 months, have you been bothered by:	Never, Once/a few times per year, Once/a few times per month, Several times/week, Every day	**Sleep disturbance**: Variables based on Keklund and Akerstedt’s work [28] were combined into one variable based on the mean of the four items, rescaled to a 0–10 scale, were 0 is a low level of sleep disturbance and 10 is a high level of sleep disturbance.
		– difficulties falling asleep?		
		– repeated awakenings with difficulties going back to sleep?		
		– not being thoroughly rested at awakening?		
		– feeling tired/sleepy during studies or leisure time?		
	**Stress** (Mood)	How have you felt during studies/work in the past 7 days?	Not at all, Almost, A bit, Quite, Very, Extremely	Based on the Mood scale [29]. Low stress or energy = 1 and high stress or energy = 6.
		– rested		
		– tense		
		– stressed		
		– relaxed		
		– pressured		
		– calm		
	**Energy** (Mood)	How have you felt during studies/work in the past 7 days?	Not at all, Almost, A bit, Quite, Very, Extremely	
		– active		
		– listless		
		– energetic		
		– ineffective		
		– alert		
		– passive		
General performance	**Decreased general performance**	Have pain/aches in the muscles/joints affected your performance in general during the last month?	Yes, No	1 = yes
				0 = no

Variable names written in bold are the variables used in the analyses.

Rasch analysis was performed on the items in the *sleep disturbance* variable [28] to check the relevance of combining the items into one single sleep disturbance score [30]. The summary statistics from this analysis were as follows: item fit: mean = 0.0 and standard deviation (SD) = 1.3; person fit: mean = 0.4 and SD = 1.3, which is good because a mean of 0 and an SD of 1.0 indicate optimal fit. Separation index = 0.70, which is acceptable. There were no reversed thresholds among the possible answer categories; hence, all possible answer categories were informative and used by the respondents in the order intended. For the variable *sleep disturbance* the mean score of the included items was calculated and *sleep disturbance* was then standardized to a 0–10 scale.

The two dimensions of mood, *stress* and *energy*, are related, to some extent, to emotional wellbeing [31]. The mood scores were originally constructed to assess mood in two dimensions in occupational surveys concerning the work environment. In the original study, stress had a neutral point of 2.4, representing neither stressed nor calm; while energy had a neutral point of 2.7, meaning neither full of energy nor passive. It should be noted that in the original phrasing, *present mood* was examined, while in this study, *mood over the past 7 days* was the focus. For the variables *stress* and *energy* the mean score of the included items was calculated, according to original use of the scores [29,32].

As explained above the variables *sleep disturbance*, *stress*, and *energy* were constructed by combining the separate items into variables and were treated as numerical in the analysis. The decision to treat these three variables as numerical is based on results from Rasch analysis. When data fit the Rasch model, a linear transformation of the raw ordinal score is obtained, which converts the ordinal variables into a one-dimensional assessment on the logit scales [30].

### Statistical methods

The baseline distribution of *present pain duration* described in terms of the median (md) value for the number of days with pain, and the 1^st^ and 3^rd^ quartiles (Q1 and Q3), together with the mean number of days indicated skewness: women: mean = 652, md = 60, Q1 = 5, and Q3 = 730, men: mean = 631, md = 135, Q1 = 4, and Q3 = 912. A descriptive graph is presented for the baseline data for *duration* to show the distribution of the pain duration in more detail than is later used in the analysis. The duration of pain was, in the analysis, categorized into the four categories: 1–7 days, 8–90 days, 91–365 days and more than 365 days. This choice of categories was based both on commonly used cut-offs for duration of pain and on the statistically-based requirement of enough individuals in each group.

Descriptive data and descriptive baseline analysis were performed presenting results for the health outcomes between the two groups formed by the variable *current pain*, individuals with and without current pain.

The pain assessments and the outcomes were both collected on five occasions: t = 0, 1, 2, 3, 4. Hence, the data used in the regression analyses are derived from individuals followed over 4 years. This strengthens the analyses, as comparing more homogenous units, such as examining changes in individuals, reduces the chance that there are unattended confounding factors influencing the results. Even more important in this study, this analysis will give information on within-individual difference in health outcomes when reporting and not reporting current pain. In order to handle the longitudinal design, mixed models (PROC MIXED in SAS, version 9.2; SAS Institute, Cary, NC, USA) were used to analyze the relationships between pain assessment and the outcomes *sleep disturbance*, *stress*, and *energy*. In these analyses men and women were not separately analyzed as the baseline results showed very similar results, but were included as an explanatory variable in the regression models when relevant. The models were constructed follows: the gender variable was included if p ≤ 0.05. The interaction between gender and the explanatory variable of interest was also tested, but nowhere found statistically significant.

Both the sleep disturbance score and the mood scores are each based on several items that were ordinal, but as described above Rasch analysis was performed and the resulting scores could then be analyzed using parametric methods.

The variable of general health was originally an ordinal variable, but dichotomized into a binary variable *very good health*. The percentages and numbers of respondents in each original response category for the separate items and separate ordinal categories included in *very good health* and *sleep disturbance* are presented in Table 2.

**Table 2:** **Baseline distribution of the answer categories for the separate items included in the outcome variables *very good health *and *sleep disturbance***

**Women N** = **627**	***Perceived health % *(n)**		***Little interest or pleasure in doing things % *(n)**	***Feeling down***, ***depressed***, ***or hopeless % *(n)**
Very good	32% (204)	Yes	33% (210)	40% (252)
Pretty good	50% (312)	No	67% (417)	60% (375)
Neither good	12% (73)			
Pretty poor	5% (34)			
Poor	1% (4)			
	***Difficulties falling asleep***	***Repeated awakenings with difficulties going back to sleep***	***Not thoroughly rested at awakening***	***Tired***/***sleepy during studies or leisure time***
Never,	12% (76)	34% (212)	3% (17)	1% (6)
Once/a few times per year,	35% (221)	39% (246)	13% (81)	6% (41)
Once/a few times per month,	39% (244)	21% (130)	36% (229)	40% (249)
Several times/week,	12% (76)	5% (35)	41% (256)	43% (268)
Every day	2% (10)	1% (4)	7% (44)	10% (63)
**Men N = 573**	***Perceived health % *(n)**		***Little interest or pleasure in doing things % *(n)**	***Feeling down***, ***depressed***, ***or hope***-***less % *(n)**
				
Very good	35% (201)	Yes	26% (148)	30% (170)
Pretty good	47% (269)	No	74% (425)	70% (403)
Neither good	13% (75)			
Pretty bad	5% (26)			
Bad	0.4% (2)			
	***Difficulties falling asleep***	***Repeated awakenings with difficulties going back to sleep***	***Not thoroughly rested at awakening***	***Tired***/***sleepy during studies or leisure time***
Never	19% (107)	52% (301)	4% (21)	2% (8)
Once/a few times per year,	35% (202)	33% (191)	15% (89)	13% (74)
Once/a few times per month,	33% (187)	11% (61)	37% (211)	38% (218)
Several times/week,	10% (57)	3% (15)	36% (209)	40% (231)
Every day	3% (20)	1% (5)	8% (43)	7% (42)

% = percentage of individuals answering a specific category; n = number of individuals answering a specific category.

All analyses were performed using the statistical package SAS, version 9.2 (SAS Institute, Cary, NC, USA).

For the outcome *very good health* a random intercept logistic regression was used for the longitudinal design (PROC NLMIXED in SAS, version 9.1; SAS Institute, Cary, NC, USA).

In the Additional file 1: Appendix, the parameter estimates for the regression models are presented. These results were used to calculate the results presented in the result section.

## Results

### Baseline prevalence

The baseline prevalence of *present pain* in upper back/neck was 27% among women and 11% among men. The baseline prevalence for neck pain in combination with at least one more pain site (lower back or arms/hands) was 15% for women and 5% for men and the prevalence of pain in all three sites (lower back and arms/hands) was 5% for women and 2% for men.

**Figure 3:**
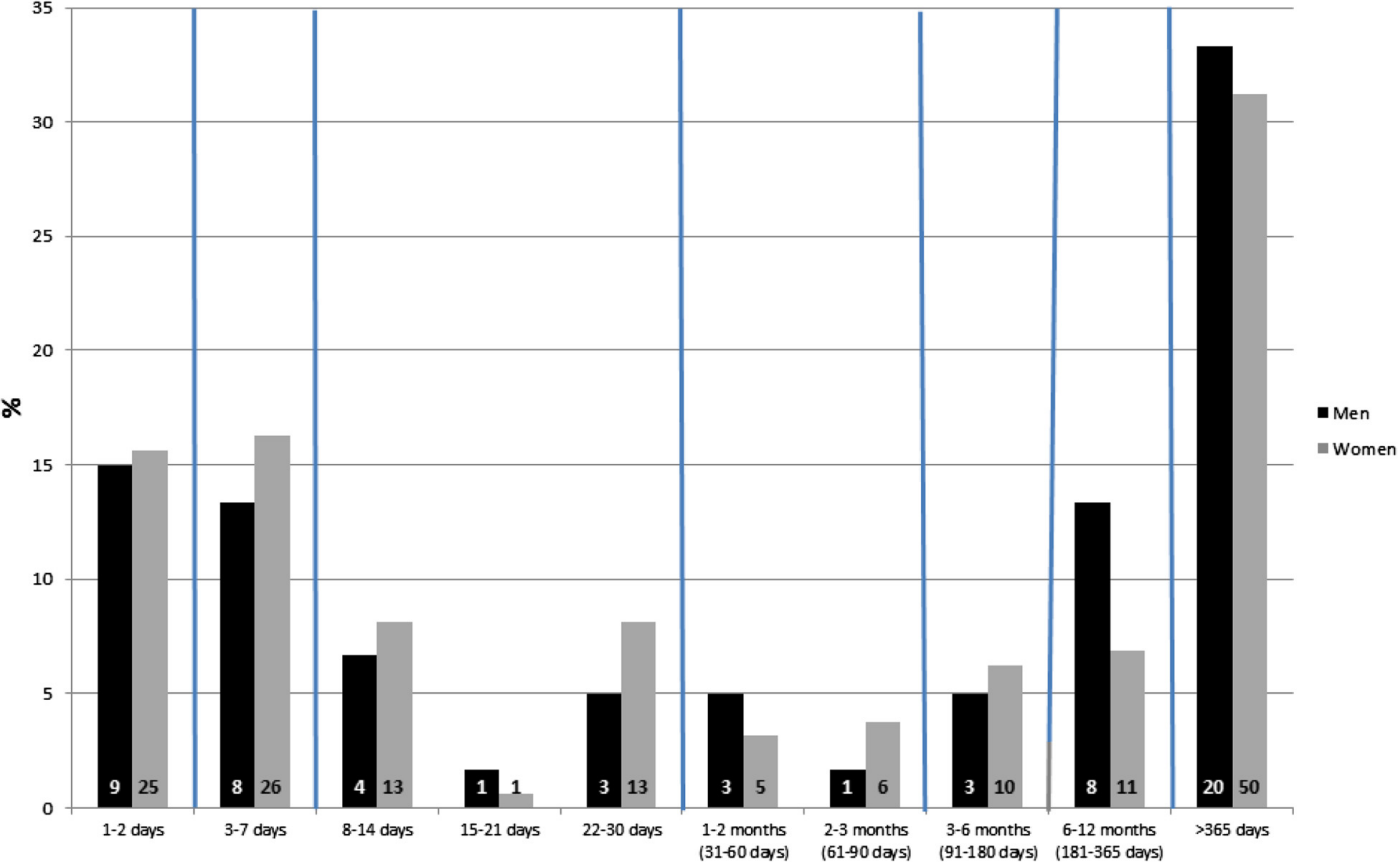
**Distribution of the duration of pain at baseline.** Note that the bars represent different length of time (weeks, months etc. denoted by the vertical lines). Inside the bars the numbers of observations are presented. Men: N = 60, Women: N = 160.

Both among men and women a large part of the neck pain represented a short duration of only 1–2 days (15%, 16%) and duration of 3–7 days (13%, 16%) (Figure 3). However, long-lasting pain, duration of more than 365 days, was also very common (men: 33%, women: 31%).

Among the participants with current neck pain at baseline, 31% had decreased general performance. Among women the percentage with decreased general performance was 32% and for men 30%.

The participants with current neck pain at baseline had lower prevalence of very good health than those without current neck pain, Table 3. The participants with current pain also had more sleep disturbance and perceived more stress, than the pain-free participants, Table 3. The results in Table 3 were similar for men and women.

**Table 3:** Baseline description of outcomes among those with and without present neck pain

	**TOTAL**		**WOMEN**		**MEN**	
	**No pain**	**Pain**	**No pain**	**Pain**	**No pain**	**Pain**
	***Prevalence, % (95% CI)***	***Prevalence, % (95% CI)***	***Prevalence, % (95% CI)***	***Prevalence, % (95% CI)***	***Prevalence, % (95% CI)***	***Prevalence, % (95% CI)***
**Very good health**	37	20	37	20	37	21
	(34.2; 40.3)	(15.4; 25.8)	(33.1; 41.9)	(14.5; 26.6)	(32.9; 41.3)	(12.5; 32.2)
	***Mean***	***Mean***	***Mean***	***Mean***	***Mean***	***Mean***
	(*95*% *CI*)	(*95*% *CI*)	(*95*% *CI*)	(*95*% *CI*)	(*95*% *CI*)	(*95*% *CI*)
**Sleep disturbance score**	4.3	5.1	4.5	5.1	4.2	5.0
	(4.22; 4.41)	(4.86; 5.31)	(4.36; 4.64)	(4.87; 5.39)	(4.01; 4.29)	(4.50; 5.40)
**Stress score**	3.3	3.9	3.4	4.0	3.1	3.6
	(3.19; 3.32)	(3.76; 4.04)	(3.28; 3.46)	(3.86; 4.19)	(3.10; 3.23)	(3.30; 3.84)
**Energy score**	3.9	3.9	4.0	3.9	3.8	3.8
	(3.87; 3.98)	(3.79; 4.01)	(3.96; 4.10)	(3.81; 4.08)	(3.76; 3.90)	(3.57; 4.0)

Presented for the total group and separately for women (N = 614–627) and men (N = 558–573).

### Longitudinal evaluation of pain questions in relation to health outcomes

In the longitudinal analysis the association of current neck pain and the health outcomes is investigated within individuals over time. For the continuous variables (not *very good health* and *decreased general performance*) within-subject and between-subject variations were achieved from the longitudinal regression analyses. The within-subject variation, regarding the health outcomes, was in all analyses larger than the between-subject variation, except for all analyses of *sleep disturbance* where the within-subject variation was smaller than the between-subject variation (see Additional file 1: Appendix).

### Association between neck pain and health outcomes

When neck pain was present the participants had lower proportion of *very good health*, higher *sleep disturbance* score, higher *stress* score and lower *energy* score, than when no neck pain was present, Table 4. Note though that the difference in *energy* score was minor, even if statistically significant.

**Table 4:** Comparing the health outcomes when neck pain was or was not present

	**No pain**	**Pain**	**Pain – No pain**	
	***Proportion*, %**	***Proportion*, %**	***Proportion diff***	***95*% *CI***
**Very good health**	26.1	11.0	−15	−19.1; -10.8
	***Mean***	***Mean***	***Mean diff***	***95*% *CI***
**Sleep disturbance score**^**a**^	4.229	4.550	0.32	0.208; 0.432
**Stress score**^**a**^	3.352	3.674	0.32	0.253; 0.391
**Energy score**^**a**^	3.998	3.934	−0.06	−0.119; -0.009

N_subj_ = 1199–1200, N_obs_ = 4281–5148. For details of regression analysis see Additional file 1: Appendix.

^a^ Adjusted for gender.

### Duration of pain in relation to health outcomes

Increasing pain duration was associated with lower proportion of very good health, higher proportion of decreased general performance and higher values on the sleep disturbance score, Table 5. The health outcomes seemed to differ most between the pain duration of 1–7 days compared to longer pain duration.

**Table 5:** **The relation between pain duration and the health outcomes**, **when neck pain was present**

**Duration**	**1-7 days**	**8-90 days**	**91-365 days**	**>365 days**	***p***-***value Type III test***
	***Proportion (95% CI)***	***Proportion (95% CI)***	***Proportion (95% CI)***	***Proportion (95% CI)***	
**Very good health**	13	6	6	8	^a^
	(8.1; 21.2)	(2.9; 11.5)	(2.5; 12.1)	(4.2; 13.7)	
**Decreased general performance**	13	30	30	30	^a^
	(8.9; 19.0)	(22.5; 39.7)	(21.4; 40.6)	(22.2; 38.8)	
	***Mean***	***Mean***	***Mean***	***Mean***	***p*-*value Type III test***
	**(*95*% *CI*)**	**(*95*% *CI*)**	**(*95*% *CI*)**	**(*95*% *CI*)**	
**Sleep disturbance score**	4.5	4.7	4.8	5.1	0.007
	(4.31; 4.76)	(4.44; 4.94)	(4.54; 5.11)	(4.83; 5.31)	
**Stress score**^**b**^	3.7	3.8	3.8	3.7	0.640
	(3.56; 3.82)	(3.65; 3.95)	(3.60; 3.93)	(3.59; 3.87)	
**Energy score**^**b**^	3.9	4.0	3.9	3.8	0.302
	(3.84; 4.04)	(3.85; 4.08)	(3.76; 4.02)	(3.73; 3.94)	

For details of regression analysis see Additional file 1: Appendix.

^a^ No type-III test is available in Proc nlmixed, but according to p-values in the Additional file 1: Appendix pain duration was statistically significant in this model. ^b^ Adjusted for gender.

N_subj_ = 480–482, N_obs_ = 794–935.

### Additional pain sites in relation to health outcomes

When additional pain sites were present the participants had lower proportion of *very good health* and higher proportion of *decreased general performance*, than when only neck pain was present. When additional pain sites were present the participants had higher *sleep disturbance* score, than when only neck pain was present, Table 6.

**Table 6:** **When neck pain was present**: **Difference in the health outcomes when having versus not having additional pain sites**

**Additional pain sites**	**No additional pain sites**	**Additional pain sites**	**Additional pain sites –No additional pain sites**	
	***Proportion (95% CI)***	***Proportion (95% CI)***	***Proportion diff***	***95% CI***
**Very good health**	12	6	−6	−12.6; 0.3
	(7.7; 18.4)	(3.7; 10.4)		
**Decreased general performance**	15	33	18	9.8; 26.1
	(11.2; 20.5)	(27.0; 40.3)		
	***Mean***	***Mean***	***Mean diff***	***95*% *CI***
	(*95*% *CI*)	(*95*% *CI*)		
**Sleep disturbance score**	4.6	4.9	0.35	0.124; 0.571
	(4.41; 4.78)	(4.75; 5.13)		
**Stress score**^**a**^	3.7	3.8	0.11	−0.024; 0.235
	(3.59; 3.79)	(3.68; 3.91)		
**Energy score**^**a**^	3.9	3.9	−0.06	−0.140; 0.065
	(3.85; 4.01)	(3.81; 3.98)		

Additional pain sites could be lower back and/or arms, hands. N_subj_ = 496–498, N_obs_ = 834–982. For details of regression analysis see Additional file 1: Appendix.

^a^ Adjusted for gender.

### Decreased general performance in relation to health outcomes

When neck pain was present sleep disturbance and stress was positively associated with pain-related decreased general performance (Table 7), but neck pain was not associated with energy. Very good health was negatively associated with pain-related decreased general performance.

**Table 7:** **When neck pain was present**: **Comparing the health outcomes when decreased general performance was or was not present**

**Decreased general performance**	**No decreased performance**	**Decreased performance**	**Decreased gen**. **performance –No decreased gen**. **performance**	
	***Proportion*, %**	***Proportion*, %**	***Proportion diff***	***95*% *CI***
**Very good health**	10.9	4.3	−7	−12.6; -1.0
	***Mean***	***Mean***	***Mean diff***	***95*% *CI***
**Sleep disturbance score**	4.623	5.101	0.48	0.243; 0.713
**Stress score**^**a**^	3.674	3.890	0.22	0.081; 0.352
**Energy score**^**a**^	3.940	3.850	−0.09	−0.198; 0.018

For details of regression analysis see Additional file 1: Appendix.

^a^ Adjusted for gender.

N_subj_ = 496–498, N_obs_ = 834–982.

## Discussion

Presence of neck pain, determined from the answer to a simple neck pain question, was related to a reduced general health, more sleep disturbance, higher stress, lower energy, and decreased general performance, among our cohort of young university students. At the same time, it appears that a large proportion of the neck pain reported among the young university students in this cohort did not seriously affect their health and perceived general performance. In the literature, the possibility has been discussed that individuals, especially in modern society, are actually reporting discomfort rather than pain [33]. This is contradicted by a study comparing the prevalence of musculoskeletal complaints in a native population living under primitive conditions to a representative sample of the Norwegian population [34]. From this, the conclusion can be drawn that the high prevalence of, for example, musculoskeletal complaints is not specific for industrialized societies. However, the question remains how severe the pain reported in the prevalence studies is, in general.

The raw measures of prevalence of pain, at baseline and at the four annual follow-ups, were 2 to 3 times higher for women than for men. This higher prevalence among women compared to men is consistent with the literature. Despite the difference in level of prevalence, the relation between current neck pain and the health outcomes were similar for women and men. Therefore separate analysis for women and men were not performed in the present study, even though this is sometimes advocated [35,36]. Hence the analyses were instead, when relevant, adjusted for women and men.

The within-subject variation, regarding the health outcomes, was in all analyses larger than the between-subject variation, except for all analyses of *sleep disturbance* where the within-subject variation was smaller than the between-subject variation. This may indicate that, except for *sleep disturbance*, knowing about the presence or not of pain explains more of the variation in health between individuals, than within individuals. Within an individual, other aspects may have to be considered to explain a substantial part of the variation in the health outcomes over time.

Most validations of the self-reported pain questions in the NQ use specific clinical diagnosis as a gold standard. This is a confusing criterion for validity as the perception of the patient is the gold standard, according to the widely-accepted pain definition (International Association for the Study of Pain, IASP), and also because the NQ was never intended to measure clinical diagnoses, as mentioned previously. Therefore, validation of pain assessments should include comparisons with other measures of the intended characteristics of pain. According to the work of the Task Force on Neck Pain and Its Associated Disorders, diagnostic procedures for non-traumatic neck pain have not been proven valid or useful [37]. They conclude that self-assessment questionnaires, if reliable and valid, are useful in management and prognosis [38].

Another qualitative requirement of pain assessment instruments is repeatability. For the NQ, repeatability was found to be good (percentage agreement = 83–90%, kappa coefficient = 0.64–0.78) when the time interval between questionnaires was about 1 week [21].

Assessing pain as the presence of pain in combination with its effect on performance increased the validity of some of our outcomes. However, because the wording *decreased general performance* may be too narrow a concept when looking at how pain affects life, a question directed at the broader effects of pain on life may increase the validity of pain assessments even further.

The reported pain in the present young age group may be less severe than, and may not affect life in the same way as, reported pain in older age groups. This could possibly explain the less clear differences in the outcomes between pain and no pain, and may point to the necessity of an additional question, as discussed previously. A ‘yes’ from a not so affected individual is not possible to distinguish from a ‘yes’ from a more affected individual, which may explain the high prevalence of pain in such a young age group.

Epidemiological studies regarding pain outcomes could benefit from more clearly specified definitions of those aspects of pain that are of most interest and importance for the individual, workplace or society.

### Limitations

There is a risk of bias due to homogeneity of answers when assessments are self-reported; for example, when a respondent gives a negative answer to one health item, there is a tendency for him or her to also report negatively about other health aspects. This type of bias could be decreased to some extent if an outcome is related both to pain assessments made at the same time as the outcome, and to assessments from the previous year, as done in the present study.

The results of this study should be generalized with caution, because the respondents, being young university students enrolled in academic studies, form a group with quite specific characteristics.

At the time of the study, it was not possible to include figures in the web-based questionnaire and hence, only words were used to define the location upper back or neck. This could endanger the validity of the pain location ‘upper back/neck’ referred to in the questionnaire. To validate the pain questions regarding pain location medical examinations were conducted on a sub-sample of 42 participants from the baseline of the university cohort. These medical examinations included pain drawings from which presence of neck pain could be defined and compared with the answers from the questionnaire. From these drawings, the agreement between neck pain, according to Task Force on Neck Pain and Its Associated Disorders, and pain in the upper back and neck (Figure 2) was 93% (95% CI 81.0; 97.5). The three participants not in agreement with the definition of the pain area were defined as having pain in the upper back, according to Figure 2, but the marked region was not wholly included in the area defined by the Task Force on Neck Pain and Its Associated Disorders. All pain drawings showing areas outside (and not even partly included in) the area defined by the Task Force on Neck Pain and Its Associated Disorders were clearly separated from this, e.g., low back, hands or knees. Hence, none of the 42 participants had a problem answering the question due to lack of a picture.

Asking respondents questions about the exact number of days of past pain can result in recall bias. This could possibly explain why the probability of decreased general performance differed between the first category of *duration of present pain* and other categories, but those other categories did not differ between each other. On the other hand, this could also be explained by a threshold effect. It has been suggested in the literature that to avoid possible recall bias, respondents should probably not be asked for details regarding duration of pain that occurred more than approximately 3 months ago [39,40]. We recommend the use of a scale with broader categories of duration if an assessment needs to consider duration of pain over long time periods.

In this paper, the sleep disturbance variables were combined into one variable through Rasch analysis. The advantage of this method is that linear regression would be appropriate; the disadvantage is that in this study, it is hard to interpret the size (in terms of “clinical” importance) of a possible difference in sleep disturbance, between those reporting and those not reporting sleep disturbance.

## Conclusions

This study of young university students has demonstrated that simple neck pain survey questions capture features of pain that affect aspects of health such as perceived general health, sleep disturbance, mood in terms of stress and energy. However, simple pain questions are mainly useful for group descriptions rather than for describing or following pain in an individual, as knowing about the presence or not of pain explains more of the variation in health between individuals, than within individuals.

## Competing interests

The authors declare that they have no competing interests.

## Authors' contribution

A.E. performed the statistical analysis, interpreted the results, and wrote the main part of the text. M.H. was in charge of the design and acquisition of data, made substantial intellectual contributions to the study, and was involved in drafting the manuscript and revising it critically. Both authors have read and approved the final manuscript.

## Supplementary Material

Additional file 1 **Appendix.** The parameter estimates presented in Table A, were used to calculate the results in Table 7 in the result section. The parameter estimates presented in Table B, were used to calculate the results presented in Tables 4 and 5 in the results section. The parameter estimates presented in Table C, were used to calculate the results presented in Table 6 in the results section. Click here for file
