# Supplementary material for: Simple neck pain questions used in surveys, evaluated in relation to health outcomes: a cohort study
Source: BMC Res Notes. 2012 Oct 26;5:587. doi: 10.1186/1756-0500-5-587 (PMC3538694; doi:10.1186/1756-0500-5-587)
Supplement: Additional file 1 — Appendix. The parameter estimates presented in Table A, were used to calculate the results in Table 7 in the result section. The parameter estimates presented in Table B, were used to calculate the results presented in Tables 4 and 5 in the results section. The parameter estimates presented in Table C, were used to calculate the results presented in Table 6 in the results section. [file 1756-0500-5-587-S1.doc]

Table A. Longitudinal analysis. Results from regression analyses using mixed models (Proc Nlmixed and Proc Mixed, SAS Vers 9.2)

| **Comparing health outcomes when current neck pain is present and not** | **TOTAL** | | **When current neck pain is present: Comparing health outcomes when general performance is reduced or not due to current pain** | **TOTAL** | |
| --- | --- | --- | --- | --- | --- |
| N_subj_ = 1199-1200, N_obs_ = 4281-5148 | Estimates | p-value | N_subj_ = 496-498, N_obs_ = 834-982 | Estimates | p-value |
| Very good health(t)^a^ | −1.04 | <0.001 | Very good health(t)^a^ | −2.10 | <0.001 |
| *Intercept* | −1.05 | <0.001 | *Intercept* | −1.01 | 0.0011 |
| *Current neck pain(t)* Inter-individual variance | 4.28 | <0.001 | *Current neck pain(t) and DP* Inter-individual variance | 4.33 | 0.0009 |
| Sleep disturbance(t) | 4.72 | <0.001 | Sleep disturbance(t) | 5.10 | <0.001 |
| *Intercept* | 0.35 | <0.001 | *Intercept* | 0.48 | <0.001 |
| *Women Current neck pain(t)* Inter-individual variance Intra-individual variance | 0.32 | <0.001 | *Current neck pain(t) and DP* Inter-individual variance Intra-individual variance | 1.71 |  |
|  |  |  |  | 1.38 |  |
|  | 1.54 |  |  |  |  |
|  | 1.23 |  |  |  |  |
| Stress(t) | 3.80 | <0.001 | Stress(t) | 4.05 | <0.001 |
| *Intercept* | 0.25 | <0.001 | *Intercept* | 0.32 | <0.001 |
| *Women* | 0.32 | <0.001 | *Women* | 0.22 | 0.002 |
| *Current neck pain(t)* | 0.34 |  | *Current neck pain(t) and DP* | 0.40 |  |
| Inter-individual variance | 0.62 |  | Inter-individual variance | 0.65 |  |
| Intra-individual variance |  |  | Intra-individual variance |  |  |
| Energy(t) | 4.02 | <0.001 | Energy(t) | 3.92 | <0.001 |
| *Intercept* | 0.17 |  | *Intercept* | 0.13 |  |
| *Women* | −0.06 |  | *Women* | 0.09 |  |
| *Current neck pain(t)* | 0.20 |  | *Current neck pain(t) and DP* | 0.20 |  |
| Inter-individual variance | 0.40 |  | Inter-individual variance | 0.44 |  |
| Intra-individual variance |  | <0.001 | Intra-individual variance |  | 0.047 |
|  |  | 0.0227 |  |  | 0.101 |

^a^Random intercept logistic regression

**Table B.** N_subj_ = 480-482, N_obs_ = 794-935

| **When current neck pain is present: Health outcomes in relation to duration of neck pain** | **TOTAL** | |
| --- | --- | --- |
|  | Estimates | p-value |
| Very good health(t)^a^ | −1.87 | <0.001 |
| *Intercept* | 0 |  |
| *1*–*7 days* | −0.90 | 0.010 |
| *8*–*90 days* | −0.96 | 0.021 |
| *91*–*365 days* | −0.61 | 0.070 |
| *>365 days* | 4.49 |  |
| Inter-individual variance |  |  |
| Decreased general performance(t)^a^ | −1.89 | <0.001 |
| *Intercept* | 0 |  |
| *1*–*7 days* | 1.06 | <0.001 |
| *8*–*90 days* | 1.05 | <0.001 |
| *91*–*365 days* | 1.04 | <0.001 |
| *>365 days* | 2.02 |  |
| Inter-individual variance |  |  |
| Sleep disturbance(t) | 4.83 | <0.001 |
| *Intercept* | −0.29 |  |
| *1*–*7 days* | −0.14 | 0.007 |
| *8*–*90 days* | 0 |  |
| *91*–*365 days* | 0.24 |  |
| *>365 days* | 1.71 |  |
| Inter-individual variance | 1.43 |  |
| Intra-individual variance |  |  |
| Stress(t) | 3.87 | <0.001 |
| *Intercept* | 0.27 |  |
| *Woman* | −0.04 | 0.002 |
| *1*–*7 days* | 0.07 | 0.635 |
| *8*–*90 days* | 0.03 |  |
| *91*–*365 days* | 0 |  |
| *>365 days* | 0.40 |  |
| Inter-individual variance | 0.65 |  |
| Intra-individual variance |  |  |
| Energy(t) | 3.90 | <0.001 |
| *Intercept* | 0.12 |  |
| *Woman* | 0.10 | 0.066 |
| *1*–*7 days* | 0.13 | 0.302 |
| *8*–*90 days* | 0.05 |  |
| *91*–*365 days* | 0 |  |
| *>365 days* | 0.19 |  |
| Inter-individual variance | 0.46 |  |
| Intra-individual variance |  |  |

**Table C.** N_subj_ = 496-498, N_obs_ = 834-993

| **When current neck pain is present: Health outcomes in relation to additional pain sites** | **TOTAL** | |
| --- | --- | --- |
|  | Estimates | p-value |
| Very good health(t)^a^ | −1.99 | <0.001 |
| *Intercept* | −0.71 |  |
| *Additional pain sites* | 3.94 | 0.006 |
| Inter-individual variance |  |  |
| Decreased general performance(t)^a^ | −1.71 | <0.001 |
| *Intercept* | 1.02 |  |
| *Additional pain sites* | 2.03 | <0.001 |
| Inter-individual variance |  |  |
| Sleep disturbance(t) | 4.60 | <0.001 |
| *Intercept* | 0.35 |  |
| *Additional pain sites* | 1.78 | 0.002 |
| Inter-individual variance | 1.36 |  |
| Intra-individual variance |  |  |
| Stress(t) | 3.53 | <0.001 |
| *Intercept* | 0.31 |  |
| *Woman* | 0.11 | <0.001 |
| *Additional pain sites* | 0.41 | 0.111 |
| Inter-individual variance | 0.65 |  |
| Intra-individual variance |  |  |
| Energy(t) | 3.86 | <0.001 |
| *Intercept* | 0.14 |  |
| *Woman* | −0.04 | 0.043 |
| *Additional pain sites* | 0.21 | 0.471 |
| Inter-individual variance | 0.44 |  |
| Intra-individual variance |  |  |
